# Kawasaki Disease following administration of 13-valent pneumococcal conjugate vaccine in young children

**DOI:** 10.1038/s41598-019-51137-5

**Published:** 2019-10-11

**Authors:** Chee Fu Yung, Xiangmei Ma, Yin Bun Cheung, Bee Khiam Oh, Sally Soh, Koh Cheng Thoon

**Affiliations:** 1 Infectious Disease Service, KK Women’s and Children’s Hospital, 100 Bukit Timah Road, 229899 Singapore, Singapore; 2 Lee Kong Chian School of Medicine, Nanyang Technological University, 11 Mandalay Road, 308232 Singapore, Singapore; 3 Duke-NUS Medical School, 8 College Road, 169857 Singapore, Singapore; 4 Centre for Quantitative Medicine, Duke-NUS Medical School, Singapore, Singapore; 5 Tampere Center for Child Health Research, University of Tampere and Tampere University Hospital, Tampere, Finland; 6 Vigilance Branch, Health Products Regulation Group, Health Sciences Authority, Singapore, Singapore; 7 Yong Loo Lin School of Medicine, National University of Singapore, 1E Kent Ridge Road, National University Health System Building (NUH), 119228 Singapore, Singapore

**Keywords:** Paediatric research, Risk factors

## Abstract

Kawasaki disease (KD) is a systemic vasculitis mainly affecting young children and the leading cause of acquired heart disease in developed countries. We performed a self-controlled case series analysis to investigate the association between PCV13 and KD. All hospitalized KD cases <2 y old from our hospital in Singapore from 2010 to 2014 were included. Complete KD cases were classified based on the definitions of the American Heart Association. During the study period, 288 KD cases were identified. A total of 21 KD cases (12 were classified as Complete KD) had date of onset within the risk interval of day 1 to day 28 post PCV13. The age-adjusted Relative Incidence (RI) for KD following PCV13 dose 1, dose 2 and dose 3 were 1.40 (95%CI, 0.72 to 2.71), 1.23 (95% CI, 0.62 to 2.44) and 0.34 (95% CI, 0.08 to 1.40) respectively. There were seven Complete KD cases with onset during the risk interval after dose 1 of PCV13 (age-adjusted RI 2.59, 95%confidence interval (CI), 1.16 to 5.81). We did not detect a significant increased risk for overall KD among PCV13 recipients. However, a significant association between PCV13 and Complete KD was noted following receipt of the first dose of PCV13.

## Introduction

Kawasaki disease (KD) is a systemic vasculitis characterised by specific clinical features mainly affecting infants and young children. Diagnosis is based primarily on clinical criteria as there is no specific confirmatory laboratory test available^1^. It can trigger changes to the coronary arteries (approximately 25% of untreated KD) in some children resulting in death or long term morbidity. However, the risk of coronary artery aneurysm (CAA) can be reduced with prompt immunoglobulin treatment. The aetiology of KD remains unknown although it is now the leading cause of acquired heart disease amongst children in North America, Europe and Japan^1^. KD has been reported worldwide but the epidemiology varies significantly between countries or regions. Reported incidences from North-Asian countries such as Japan and Korea are 10–20 times higher than incidences from USA and Europe^2^.

A 7-valent pneumococcal conjugate vaccine (PCV7) (Prevenar; Wyeth) and subsequently a 13-valent pneumococcal conjugate vaccine (PCV13) (Prevenar 13; Pfizer) was licensed by the Food and Drug Administration (FDA) in 2000 and 2010 respectively. Implementation of public health Pneumococcal conjugate vaccine (PCV) immunization programmes, particularly in developed countries have led to substantial reduction in diseases such as pneumonia, bacteremia and meningitis caused by *S. pneumonia* infection^3,4^. Currently, PCV13 is recommended as a 4 dose schedule, administered at 2, 4, 6 months with a booster at 12–15 months by the Advisory Committee on Immunization Practices (ACIP)^5^. In some countries such as the United Kingdom, a 3 dose schedule at 2, 4 and 12–15 months is recommended. In Singapore, PCV7 was licensed in 2002 and PCV13 since 2010. Pneumococcal conjugate vaccine was formally recommended in the national childhood immunisation schedule by the Ministry of Health in 2010 as a 3 dose schedule at 3 months followed by a second dose at 5 months and booster at 12 months. However, the licensed 4 dose schedule option similar to the ACIP is also used especially by private healthcare providers.

To date, only a handful of published studies have investigated the link between PCV and KD. A phase IV database analysis by Center *et al*. reported an increased risk of KD hospitalizations amongst recipients of PCV7 compared to historical controls (unadjusted RR 2.02 (95% CI, 1.16 to 3.63)^6^. However, after controlling for possible confounders including sex, race, age at first dose, etc., the RR of KD hospitalization was no longer statistically significantly (RR 1.67; 95% CI, 0.93 to 3.00)^6^. Tseng *et al*. compared risk of KD between recipients of PCV13 versus PCV7 but did not find a statistically significant increase in risk although the point estimate RR was 1.94^7^. An evaluation of 29 KD cases for PCV7 reported to the Vaccine Adverse Event Reporting System (VAERS) in USA up to 2007 did not find any increased risk^8^. In another study, recipients of PCV13 were found to have an elevated but statistically non-significant risk of KD in the 28 days following immunization compared to historical controls who were recipients of PCV7 (RR 1.94, 95%CI, 0.79 to 4.86)^7^. More recently, a report from the Sentinel initiative, Center for Biologics Evaluation and Research (CBER) using 87 confirmed KD cases in a self-controlled risk interval analysis identified no elevated risk of KD amongst PCV13 recipients (relative risk (RR) 1.07, 95%CI, 0.70 to 1.63)^9^. In the same report, a cohort analysis method composed of 80 potential KD cases (based on claims) in the 1 to 28 days risk window also found no elevated risk of KD (RR 0.84, 95%CI, 0.65 to 1.08).

In this paper, we aim to investigate the association between PCV13 and KD using the self-controlled case series (SCCS) study design^10^. Our apriori null hypothesis stated that the risk of KD within the 28 day risk interval following receipt of any PCV13 dose was the same as control periods outside the risk window.

## Methods

### Study setting

KK Women’s and Children’s Hospital (KKH) is the single largest public healthcare specialist women’s and children’s hospital in Singapore. The hospital has approximately 800 beds and is the site of an ongoing active surveillance vaccine safety programme set up in 2009 in collaboration with the Health Sciences Authority (Singapore’s regulatory authority on medical products). Details of this active surveillance system were previously described^11^. Briefly, all paediatric admissions to the hospital are prospectively screened for a specific range of diseases and syndromes including KD. Detailed clinical and laboratory information are collected together with immunization history for the purpose of monitoring safety in childhood immunizations.

### Data sources and definitions

We extracted all hospitalized cases of KD in our surveillance database from 1^st^ January 2010 to 31^st^ December 2014. To ensure complete extraction of all KD cases admitted to KKH, we also screened the hospital discharge database for KD cases using International Classification of Diseases (ICD), Ninth Revision and Tenth Revision KD codes 446.1 and M30.3 respectively, in any diagnostic field. The immunization history of each case was obtained from the child’s health information booklet. In addition, the patient’s identifiers and unique national identity number was linked with the National Immunisation Registry (NIR) which records childhood immunizations administered by all private and public healthcare providers in Singapore. Ethics approval was obtained from the SingHealth Centralized Institutional Review Board, Singapore.

For the purpose of this study, only cases with age of onset <2 years and children who were citizens or permanent residents of Singapore were included in the study. Cases who had ever received PCV7 were excluded. We classified KD cases based on the definitions of the American Heart Association (AHA) KD Scientific Statement as shown in Table 1. Complete KD was defined as five or more days of fever AND evidence of at least four of the five main clinical manifestations. This was similar to the level 1a diagnostic certainty criteria developed by the Brighton Collaboration except that their criteria require only four days of fever^12^. Incomplete KD were defined as cases with prolonged unexplained fever AND < 4 of the five main clinical manifestations (Table 1).

**Table 1:** Case definitions for Complete KD and Incomplete KD.

Complete KD	Incomplete KD
Fever ≥ 5 days	Fever ≥ 4 days
AND	AND
At least 4 of the following 5 clinical features:	At least 2 or 3 of the 5 clinical features:
- Oromucosal: Erythema; Crusting of Lips; Starwberry Tongue	- Oromucosal: Erythema; Crusting of Lips; Starwberry Tongue
- Conjunctival injection	- Conjunctival injection
- Polymorphous skin rash	- Polymorphous skin rash
- Extremeties: Induration of hands and feet; Peeling of skin	- Extremeties: Induration of hands and feet; Peeling of skin
- Cervical lymphadenopathy (≥1.5 cm diameter)	- Cervical lymphadenopathy (≥1.5 cm diameter)

### Statistical analysis

We used the SCCS method, which used case patients only to compare the chance of an outcome occurring in fixed intervals of time after immunization relative to unexposed times^10,13^. The observation period was from 1^st^ January 2010 to 31^st^ December 2014. Risk intervals after each PCV13 dose were defined as day 1 to day 28 following receipt of the vaccine with day 0 being the day of immunization. All other times during the observation period were defined as ‘unexposed’ periods (control interval). We used the documented date of fever onset as the date of onset of KD. KD cases with fever onset on day of PCV13 immunization (day 0) were excluded from analysis.

Relative incidence (RI) of KD during the risk interval compared to the control interval was estimated using conditional Poisson regression^10^. Separate stratified analysis for Complete KD and Incomplete KD were also performed. In view of the known varying incidence of KD by age, for our primary analysis we adjusted for age in the model using four predetermined age categories: 0 m to <6 m, 6 m to <12 m, 12 m to <18 m, 18 m to <24 m. The primary analysis included all eligible KD cases regardless of PCV vaccination status as information from unvaccinated cases facilitated estimation and controlling of age effect. We also performed additional sensitivity analysis using monthly age interval as a cubic spline function with one knot at 12 months old as well as using vaccinated cases only^14^. Although the risk of KD is known to be higher in males, the SCCS negated the need to control for such fixed confounders. The two-sample t-test was used for comparison of continuous variables between groups, and the Fisher’s exact test was used for categorical variables. Statistical analysis were two-tailed and P < 0.05 was considered significant. All statistical analysis were performed using Stata (Version 13.1).

Ethical approval was granted from the SingHealth Centralise Institutional Review Board (Reference Number: 2012/340/E). All research was performed in accordance with relevant guidelines/regulations. Waiver of informed consent was granted by the ethics committee. The study did not involve any experiments on humans and/or the use of human tissue samples.

## Results

### Kawasaki disease cases

**Figure 1:**
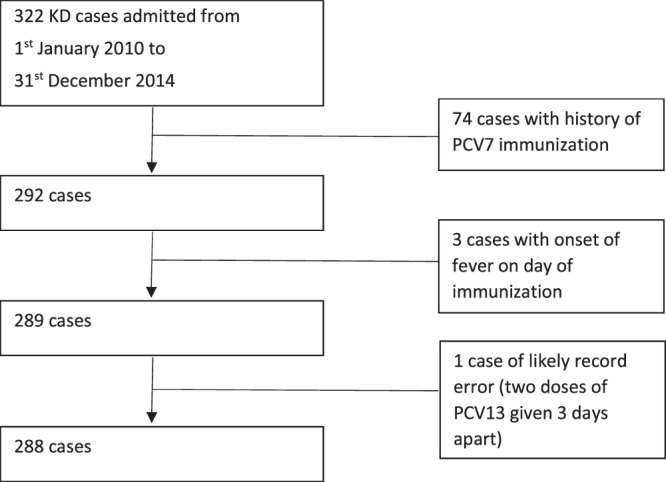
Consort flow diagram of criteria for case inclusion and exclusion.

From 1^st^ January 2010 to 31^st^ December 2014, 366 KD cases <2 years old were admitted to KK Hospital. From these, we excluded 74 cases with a positive history of PCV7 immunization, three cases with onset of fever/KD on the day of immunisation and one case with a likely PCV13 immunisation date record error (only three days between dose 1 and dose 2) (Fig. 1). Among the final 288 KD cases used in the analysis, 172 (60%) were Complete KD cases. There were slightly more male cases (57%) and 192 (66%) had a positive history of PCV13 immunization. As shown in Table 2, there were no major differences between KD cases in terms of PCV13 status for various demographic and clinical characteristics: age at presentation, gender, length of hospital admission and days of fever prior to admission. Only the ethnic distribution between the two groups were found to be statistically significantly different (p = 0.018).

**Table 2:** Characteristics of participants and KD disease episodes, by PCV13 vaccination status and stratification for Complete KD or Incomplete KD.

Variables	Unvaccinated (n = 96)	KD	*P* value*	Unvaccinated (n = 61)	Complete KD	*P* value*	Unvaccinated (n = 35)	Incomplete KD	*P* value*
		Vaccinated (n = 192)			Vaccinated (n = 111)			Vaccinated (n = 81)	
**Age at Admission (month)**									
Mean	12.24	11.32	0.228	13.12	12.65	0.597	10.69	9.50	0.352
Median	12.29	10.53		12.65	11.93		9.99	7.82	
Range	1.35–23.52	1.58–23.89		2.86–23.52	2.53–23.52		1.35–23.13	1.58–23.89	
**Sex**									
Male	55 (57.29%)	109 (56.77%)	1.000	34 (55.74%)	63 (56.76%)	1.000	21 (60.00%)	46 (56.79%)	0.839
Female	41 (42.71%)	83 (43.23%)		27 (44.26%)	48 (43.24%)		14 (40.00%)	35 (43.21%)	
**Ethnicity**									
Chinese	63 (65.63%)	152 (79.17%)	0.018	41 (67.21%)	86 (77.48%)	0.247	22 (62.86%)	66 (81.48%)	0.065
Malay	23 (23.96%)	23 (11.98%)		14 (22.95%)	14 (12.61%)		9 (25.71%)	9 (11.11%)	
Indian	6 (6.25%)	5 (2.60%)		3 (4.92%)	3 (2.70%)		3 (8.57%)	2 (2.47%)	
Others	4 (4.17%)	12 (6.25%)		3 (4.92%)	8 (7.21%)		1 (2.86%)	4 (4.94%)	
**Total Length of Stay**									
Mean	4.99	5.15	0.572	4.79	4.73	0.854	5.34	5.72	0.476
Median	5	4		4	4		5	5	
Range	2–12	2–24		2–11	2–14		2–12	2–24	
**Days of Fever before Admission**									
Mean	3.56	4.09	0.069	3.70	3.92	0.510	3.31	4.33	0.048
Median	3	4		4	4		3	4	
Range	0–14	0–21		0–14	0–15		0–6	0–21	

*Fisher’s exact test is used for categorical variables; two-sample t-test for quantitative variables.

### Risk of kawasaki disease following PCV13 immunization

**Figure 2:**
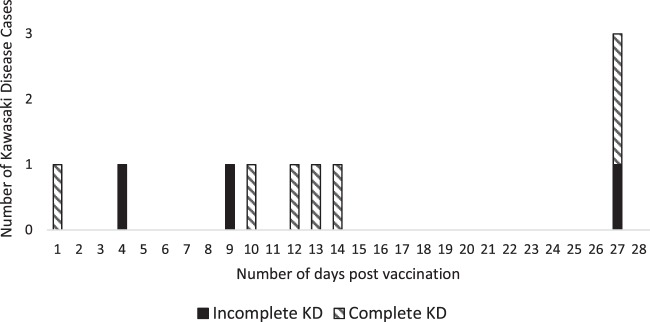
Temporal distribution of KD cases within 28 days of dose 1 PCV13 vaccination

A total of 21 KD cases had date of onset within the risk interval of day 1 to day 28 post PCV13 immunization (Table 3). Amongst the 21 KD cases 12 were classified as Complete KD and 9 were Incomplete KD. There were 10, 9, 2 and 0 KD cases with date of onset within the risk interval after PCV13 dose 1, dose 2, dose 3 and dose 4 respectively (Fig. 2). The age-adjusted RI for KD following PCV13 dose 1, dose 2 and dose 3 were 1.40 (95%CI, 0.72 to 2.71), 1.23 (95% CI, 0.62 to 2.44) and 0.34 (95% CI, 0.08 to 1.40) respectively (Table 4). All KD cases in our cohort with onset within the risk interval received Intravenous Immunoglobulin (IVIG) and aspirin treatment. None had evidence of coronary artery dilatation or aneurysm in their final echocardiogram.

**Table 3:** Number of Kawasaki disease episodes during risk periods by PCV13 dose.

Actual dose number of PCV received	KD	Complete KD	Incomplete KD
Children received 1^st^ dose	188	109	79
Episodes within 1^st^ dose’s risk period	10	7	3
Children received 2^nd^ dose	165	95	70
Episodes within 2^nd^ dose’s risk period	9	5	4
Children received 3^rd^ dose	126	82	44
Episodes within 3^rd^ dose’s risk period	2	0	2
Children received 4^th^ dose	19	11	8
Episodes within 4^th^ dose’s risk period	0	0	0

**Table 4:** Age-adjusted Relative Incidence (RI) of KD following PCV13 by dose.

	KD		Complete KD		Incomplete KD	
	Age-adjusted RI (95% CI)	*P* value	Age-adjusted RI (95% CI)	*P* value	Age-adjusted RI (95% CI)	*P* value
Exposed for Dose 1	1.40 (0.72, 2.71)	0.316	2.59 (1.16, 5.81)	0.020	0.65 (0.20, 2.12)	0.479
Exposed for Dose 2	1.23 (0.62, 2.44)	0.545	1.31 (0.52, 3.28)	0.566	1.15 (0.41, 3.17)	0.792
Exposed for Dose 3	0.34 (0.08, 1.40)	0.136	NA	NA	1.22 (0.29, 5.14)	0.786
Exposed for Dose 4	NA	NA	NA	NA	NA	NA
Age(6m- < 12 m)	1.79 (1.29, 2.50)	0.001	3.06 (1.86, 5.04)	0.000	1.06 (0.66, 1.68)	0.812
Age(12m- < 18 m)	1.67 (1.17, 2.39)	0.005	3.29 (1.96, 5.53)	0.000	0.75 (0.43, 1.30)	0.298
Age(18m-24m)	1.23 (0.83, 1.82)	0.295	2.39 (1.37, 4.16)	0.002	0.57 (0.31, 1.06)	0.077

NA: Not applicable.

Upon stratified analysis, there were seven Complete KD cases during the risk interval after dose 1 of PCV13 (age-adjusted RI 2.59, 95% confidence interval (CI), 1.16 to 5.81) (Table 4). The age-adjusted RI for Complete KD following dose 2 of PCV13 was not statistically significant (age-adjusted RI 1.31, 95% CI, 0.52 to 3.28). No Complete KD cases occurred during the risk interval after dose 3 or dose 4 of PCV13. In the Incomplete KD group, there were three Incomplete KD cases during the risk interval after dose 1 of PCV13 (age-adjusted RI 0.65, 95%CI, 0.20 to 2.12). The RI of Incomplete KD during the risk interval for subsequent PCV13 doses was also not statistically significantly higher than the control interval (Table 4).

### Sensitivity analysis

We detected similar RI for KD following PCV13 immunization in the sensitivity analysis. Details reported in Supplementary Tables 1 and 2. In summary, the age-adjusted (monthly interval) RI for Complete KD within the risk interval following dose 1 of PCV13 was 2.31 (95%CI, 1.02 to 5.23). When only vaccinated cases (N = 111) were used for analysis, the RI for Complete KD following dose 1 of PCV13 was 2.16 (95%CI, 0.95 to 4.93). The risk of KD for subsequent doses of PCV13 were not statistically significantly elevated.

## Discussion

We did not detect a statistically significant increased risk for KD among PCV13 recipients. However, an approximate two fold increased risk of Complete KD within the 28 day risk interval following receipt of the first dose of PCV13 compared to control periods outside the risk window was noted. There is an urgent need to confirm this finding in future studies. Subsequent doses of PCV13 did not result in an increased risk of Complete KD. The results were robust in sensitivity analysis when we used monthly age-intervals or included vaccinated cases only in our models. Reassuringly, all KD cases with onset within the risk interval were treated (Intravenous Immunoglobulin (IVIG) and aspirin) and none had evidence of coronary artery sequelae in their final echocardiogram.

An important strength of our analysis was the use of the SCCS study design. It allowed us to mitigate fixed confounders relevant to KD such as gender and genetics. Genetic mutations have been reported to contribute to the well documented regional and ethnic variations in incidence of KD globally^15,16^. Observational study design’s inability to control for differences in genetic susceptibility to KD as well as other unknown fixed confounders could generate biased findings. To date, there is only one other self-controlled analysis on the subject as stated earlier. The investigation report by the Sentinel initiative, CBER found no statistically significant increased risk for KD among PCV13 recipients^9^. However, apart from the smaller sample size of 87 confirmed KD cases compared to our larger sample size of 288 KD cases, there were also other key differences in methodology. Firstly, in the US study, cases were identified electronically via ICD codes rather than prospectively via active case surveillance. Secondly, there was a protocol violation where KD cases with delayed hospitalization occurring 71 to 84 days after PCV vaccination would not have been captured. Thirdly, their adjustment for age was based on another database of KD cases (‘Kids’ Inpatient Database, Healthcare Cost and Utilization Project’) from 2009 while KD cases included for their analysis were recruited from 2010 to 2015. The smaller sample size and variations in methodology may account for the differing results. A cohort design by the same group also found no statistically significant association but in order to increase sample size, cases without chart verification (32% of cohort) were included. Therefore, considering the difficult challenge in diagnosing KD, the possible inclusion of non-KD cases could have been biased the results of the cohort analysis. Apart from the above report, there were no published studies directly comparing the risk of KD following PCV13 using controls who had not received PCV.

In our stratified analysis, the point estimate RI of KD following PCV13 was 2.59 for Complete KD which contrasted with the RI for Incomplete KD at 0.65. However, the Incomplete KD’s RI had a 95%CI which overlapped 1 (95%CI, 0.20 to 2.12). It is likely that this difference in risk between PCV13 and Complete KD versus Incomplete KD may be attributable to challenges in diagnosing KD due to the absence of any confirmatory laboratory test. Diagnosing Complete KD requires a more stringent criteria and hence, it was probable that the specificity would be higher in contrast to Incomplete KD. Misclassification or wrongly diagnosed KD cases in the Incomplete KD group could inadvertently negate any existing causative link between PCV13 and KD. Interestingly, vaccinated Incomplete KD cases had a duration of fever before they presented to hospital which was one day longer than unvaccinated cases (p = 0.048). This may suggest that these cases may have had less severe clinical manifestations which could make it harder to accurately diagnose KD. In contrast, there were no differences in duration of fever before hospitalization by vaccination status for the Complete KD group. Misclassification was also less likely in the Complete KD group since our case definition for Complete KD was actually stricter than the level 1a Brighton collaboration criteria. We required that patients had at least five days of fever similar to the AHA guidelines, instead of only four days. Our finding highlights the importance for future studies to differentiate Complete and Incomplete KD, preferably using standardised case definitions such as the AHA guidelines or Brighton Collaboration^12^. Earlier studies such as the study by Center *et al*. did not specify the criteria used to define KD except that it was ‘pre-specified’ in collaboration with the FDA^6^. However, mean duration of fever was reported to be six days which may suggest that Incomplete KD cases were included. Based on our study findings, this inclusion may have biased the analysis towards the null hypothesis for the association between PCV7 and KD.

A limitation of our study, similar to any study involving vaccination records, is the possible misclassification of PCV13 vaccination status. To mitigate this, we excluded subjects who may not be residing permanently in Singapore as they may have received PCV13 from abroad. We also collated vaccination histories from multiple sources: patient’s health booklet, National Immunization Registry and medical chart review. In addition, our findings were robust to sensitivity analysis using vaccinated cases only. Therefore, we believe it is unlikely that misclassification of PCV13 vaccination status could have biased our findings. We did not adjust for possible effects from concurrent vaccines given around the same time as PCV13. However, reviewing the immunization histories of the 7 Complete KD cases with onset with the first PCV13 dose risk window only identified 3 cases having received concurrent 5-in-1 vaccine (diphtheria, tetanus, pertussis, polio and *Haemophilus influenza* type b) within the prior 28 days. Two cases were vaccinated 27 days and one 14 days before onset of KD. Although there has been reports of temporal association of KD with childhood vaccinations, no causal association has been established with any childhood vaccines^12,17^. The variability of KD incidence by age could also confound the analysis of the relationship between PCV13 and KD. We adjusted for age in our primary model to mitigate this. Additional sensitivity analysis categorising age by smaller monthly units produced similar results. Finally, time varying factors such as seasonality and environmental factors which have been reported to be possible risk factors for KD, were also potential confounders which could limit our findings. However, the seasonality of KD has mainly been reported in temperate regions rather than tropical countries like Singapore, and furthermore, current evidence for a possible role of environmental factors remain inconclusive^2^.

## Conclusions

In conclusion, using a self-controlled case series design, we did not detect a statistically significant association between PCV13 and overall KD. However, there was a statistically significant association between PCV13 and Complete KD following receipt of the first PCV13 dose in children <2 years old. There was no evidence of any coronary artery sequelae following appropriate treatment for Complete KD following receipt of PCV13. Hence, clinical vigilance with early diagnosis and appropriate treatment of KD remains important. There is an urgent need for more studies in other settings to verify this signal in view of the potential impact on the risk-benefit of PCV13 immunization programmes globally.

## Supplementary information

Supplementary Tables

## Data Availability

The datasets generated during and/or analysed during the current study are available from the corresponding author on reasonable request.
