# Author Correction: Kawasaki Disease following administration of 13-valent pneumococcal conjugate vaccine in young children

**DOI:** 10.1038/s41598-021-87465-8

**Published:** 2021-04-12

**Authors:** Chee Fu Yung, Xiangmei Ma, Yin Bun Cheung, Bee Khiam Oh, Sally Soh, Koh Cheng Thoon

**Affiliations:** 1 Infectious Disease Service, KK Women’s and Children’s Hospital, 100 Bukit Timah Road, 229899 Singapore, Singapore; 2 Lee Kong Chian School of Medicine, Nanyang Technological University, 11 Mandalay Road, 308232 Singapore, Singapore; 3 Duke-NUS Medical School, 8 College Road, 169857 Singapore, Singapore; 4 Centre for Quantitative Medicine, Duke-NUS Medical School, Singapore, Singapore; 5 Tampere Center for Child Health Research, University of Tampere and Tampere University Hospital, Tampere, Finland; 6 Vigilance Branch, Health Products Regulation Group, Health Sciences Authority, Singapore, Singapore; 7 Yong Loo Lin School of Medicine, National University of Singapore, 1E Kent Ridge Road, National University Health System Building (NUH), 119228 Singapore, Singapore

Correction to: *Scientific Reports* https://doi.org/10.1038/s41598-019-51137-5, published online 11 October 2019

The original version of this Article required clarification, as the limitations of the approach were not clearly stated, and the marginal association between Complete Kawasaki Disease and the first dose of 13-valent pneumococcal conjugate vaccine was not placed into adequate context.

The text in the Abstract,

“We did not detect a significant increased risk for overall KD among PCV13 recipients. However, a significant association between PCV13 and Complete KD was noted following receipt of the first dose of PCV13.”

Now reads,

“We did not detect a significant increased risk for overall KD among PCV13 recipients.”

The text in the Discussion,

“We did not detect a statistically significant increased risk for KD among PCV13 recipients. However, an approximate two fold increased risk of Complete KD within the 28 day risk interval following receipt of the first dose of PCV13 compared to control periods outside the risk window was noted. There is an urgent need to confirm this finding in future studies. Subsequent doses of PCV13 did not result in an increased risk of Complete KD. The results were robust in sensitivity analysis when we used monthly age-intervals or included vaccinated cases only in our models. Reassuringly, all KD cases with onset within the risk interval were treated (Intravenous Immunoglobulin (IVIG) and aspirin) and none had evidence of coronary artery sequelae in their final echocardiogram.”

Now reads,

“We did not detect a statistically significant increased risk for KD among PCV13 recipients, for any of the four doses. However, an approximate two fold increased risk of Complete KD within the 28 day risk interval following receipt of the first dose of PCV13 compared to control periods outside the risk window was noted. This could be a chance finding as no adjustment was made for multiple comparisons. Of the 8 associations tested, in all but one test we found no evidence for an association between PCV13 and KD risk. Subsequent doses of PCV13 did not result in an increased risk of Complete KD. Reassuringly, all KD cases with onset within the risk interval were treated (Intravenous Immunoglobulin (IVIG) and aspirin) and none had evidence of coronary artery sequelae in their final echocardiogram.”

The text in the Conclusion,

“In conclusion, using a self-controlled case series design, we did not detect a statistically significant association between PCV13 and overall KD. However, there was a statistically significant association between PCV13 and Complete KD following receipt of the first PCV13 dose in children < 2 years old. There was no evidence of any coronary artery sequelae following appropriate treatment for Complete KD following receipt of PCV13. Hence, clinical vigilance with early diagnosis and appropriate treatment of KD remains important. There is an urgent need for more studies in other settings to verify this signal in view of the potential impact on the risk-benefit of PCV13 immunization programmes globally.”

Now reads,

“In conclusion, using a self-controlled case series design, we did not detect a statistically significant association between PCV13 and overall KD. Although post hoc subgroup analyses of Complete KD (uncorrected for multiple comparisons) suggested a marginal association (for first PCV13 dose in children < 2 years old), this may well represent a type I error. There was no evidence of any coronary artery sequelae following appropriate treatment for Complete KD following receipt of PCV13. Hence, whilst our data overall suggests that the vaccine does not pose a risk, ongoing surveillance for any adverse events (including KD) related to vaccination is warranted. Early diagnosis and appropriate treatment of KD remains important.”

This has now been corrected in the PDF and HTML versions of the Article.

