# Supplementary material for: Kawasaki Disease following administration of 13-valent pneumococcal conjugate vaccine in young children
Source: Sci Rep. 2019 Oct 11;9:14705. doi: 10.1038/s41598-019-51137-5 (PMC6788987; doi:10.1038/s41598-019-51137-5)
Supplement: Supplementary file 1 — Supplementary Tables [file 41598_2019_51137_MOESM1_ESM.docx]

**Title:** Kawasaki Disease following administration of 13-valent pneumococcal conjugate vaccine in young children.

**Authors:**

*Chee Fu Yung^1,2,3^

Xiangmei Ma^4^

Yin Bun Cheung^4,5^

Bee Khiam Oh^1^

Sally Soh^6^

Koh Cheng Thoon^1,3,7^

**Affiliations:**

1 Infectious Disease Service, KK Women's and Children's Hospital, 100 Bukit Timah Road, 229899, Singapore.

2 Lee Kong Chian School of Medicine, Nanyang Technological University, 11 Mandalay Road, 308232, Singapore.

3 Duke-National University of Singapore Graduate Medical School, 8 College Road, 169857, Singapore.

4 Centre for Quantitative Medicine, Duke-NUS Medical School, Singapore.

5 Tampere Center for Child Health Research, University of Tampere and Tampere University Hospital, Tampere, Finland.

6 Vigilance Branch, Health Products Regulation Group, Health Sciences Authority, Singapore.

7 Yong Loo Lin School of Medicine, National University of Singapore, 1E Kent Ridge Road, National University Health System Building (NUH), 119228, Singapore.

***Corresponding Author:**

Name Chee Fu Yung

Address KK Women's and Children's Hospital

100 Bukit Timah Road, 229899, Singapore.

Email [Yung.Chee.Fu@singhealth.com](mailto:Yung.Chee.Fu@singhealth.com).sg

Telephone 65-63945982

Fax 65-62917923

**Supplementary Material**

**Table 5: Age-adjusted* (monthly interval) Relative Incidence (RI) of KD following PCV13 by dose.**

|  | **KD**  **(N=288)** |  | **Complete KD (N=172)** |  | **Incomplete KD (N=116)** |  |
| --- | --- | --- | --- | --- | --- | --- |
|  | Age-adjusted RI  (95% CI) | *P* value | Age-adjusted RI  (95% CI) | *P* value | Age-adjusted RI (95% CI) | *P* value |
| Exposed for Dose 1 | 1·15 (0·59, 2·23) | 0·689 | 2·31 (1·02, 5·23) | 0·044 | 0·52 (0·16, 1·71) | 0·284 |
| Exposed for Dose 2 | 0·99 (0·49, 1·96) | 0·967 | 1·10 (0·43, 2·79) | 0·840 | 0·88 (0·31, 2·00) | 0·805 |
| Exposed for Dose 3 | 0·35 (0·09, 1·41) | 0·139 | NA | NA | 1·16 (0·28, 4·6) | 0·836 |
| Exposed for Dose 4 | NA | NA | NA | NA | NA | NA |

* Adjusted for age in monthly interval by cubic spline with a knot at 12 months.

NA: Not Applicable

**Table 6: Age-adjusted* (monthly interval) Relative Incidence (RI) of KD following PCV13 by dose for vaccinated cases only.**

|  | **KD**  **(N=192)** |  | **Complete KD (N=111)** |  | **Incomplete KD (N=81)** |  |
| --- | --- | --- | --- | --- | --- | --- |
|  | Age-adjusted RI  (95% CI) | *P* value | Age-adjusted RI  (95% CI) | *P* value | Age-adjusted RI (95% CI) | *P* value |
| Exposed for Dose 1 | 1·10 (0·56, 2·15) | 0·775 | 2·16 (0·95, 4·93) | 0·067 | 0·52 (0·16, 1·70) | 0·279 |
| Exposed for Dose 2 | 0·95 (0·48, 1·90) | 0·890 | 1·07 (0·42, 2·73) | 0·889 | 0·85 (0·30, 2·40) | 0·759 |
| Exposed for Dose 3 | 0·35 (0·09, 1·41) | 0·139 | NA | NA | 1·15 (0·27, 4·82) | 0·848 |
| Exposed for Dose 4 | NA | NA | NA | NA | NA | NA |

* Adjusted for age in monthly interval by cubic spline with a knot at 12 months.

NA: Not Applicable
